# Using machine learning to improve the diagnostic accuracy of the modified Duke/ESC 2015 criteria in patients with suspected prosthetic valve endocarditis – a proof of concept study

**DOI:** 10.1007/s00259-024-06774-y

**Published:** 2024-06-21

**Authors:** D. ten Hove, R. H. J. A. Slart, A. W. J. M. Glaudemans, D. F. Postma, A. Gomes, L. E. Swart, W. Tanis, P. P. van Geel, G. Mecozzi, R. P. J. Budde, K. Mouridsen, B. Sinha

**Affiliations:** 1 Department of Nuclear Medicine and Molecular Imaging, University of Groningen, University Medical Center Groningen, Medical Microbiology & Infection Prevention, Hanzeplein 1, Groningen, 9713 GZ The Netherlands; 2 Department of Medical Microbiology and Infection Prevention, University of Groningen, University Medical Center Groningen, Groningen, The Netherlands; 3 Biomedical Photonic Imaging group, Faculty of Science and Technology, University of Twente, Enschede, The Netherlands; 4 Department of Internal Medicine and Infectious Diseases, University of Groningen, University Medical Center Groningen, Groningen, The Netherlands; 5 Department of Cardiology, Spaarne Gasthuis, Haarlem, The Netherlands; 6 Department of Cardiology, HagaZiekenhuis, The Hague, The Netherlands; 7 Department of Cardiology, University of Groningen, University Medical Center Groningen, Groningen, The Netherlands; 8 Department of Cardiothoracic Surgery, University of Groningen, University Medical Center Groningen, Groningen, The Netherlands; 9 Department of Radiology and Nuclear Medicine, Erasmus University Medical Center, Rotterdam, The Netherlands; 10 Department of Clinical Medicine, Center of Functionally Integrative Neuroscience, Aarhus University, Aarhus, Denmark

**Keywords:** Endocarditis, Machine learning, Modified Duke/ESC 2015 criteria

## Abstract

**Introduction:**

Prosthetic valve endocarditis (PVE) is a serious complication of prosthetic valve implantation, with an estimated yearly incidence of at least 0.4-1.0%. The Duke criteria and subsequent modifications have been developed as a diagnostic framework for infective endocarditis (IE) in clinical studies. However, their sensitivity and specificity are limited, especially for PVE. Furthermore, their most recent versions (ESC2015 and ESC2023) include advanced imaging modalities, e.g., cardiac CTA and [^18^F]FDG PET/CT as major criteria. However, despite these significant changes, the weighing system using major and minor criteria has remained unchanged. This may have introduced bias to the diagnostic set of criteria. Here, we aimed to evaluate and improve the predictive value of the modified Duke/ESC 2015 (MDE2015) criteria by using machine learning algorithms.

**Methods:**

In this proof-of-concept study, we used data of a well-defined retrospective multicentre cohort of 160 patients evaluated for suspected PVE. Four machine learning algorithms were compared to the prediction of the diagnosis according to the MDE2015 criteria: Lasso logistic regression, decision tree with gradient boosting (XGBoost), decision tree without gradient boosting, and a model combining predictions of these (ensemble learning). All models used the same features that also constitute the MDE2015 criteria. The final diagnosis of PVE, based on endocarditis team consensus using all available clinical information, including surgical findings whenever performed, and with at least 1 year follow up, was used as the composite gold standard.

**Results:**

The diagnostic performance of the MDE2015 criteria varied depending on how the category of ‘possible’ PVE cases were handled. Considering these cases as positive for PVE, sensitivity and specificity were 0.96 and 0.60, respectively. Whereas treating these cases as negative, sensitivity and specificity were 0.74 and 0.98, respectively. Combining the approaches of considering possible endocarditis as positive and as negative for ROC-analysis resulted in an excellent AUC of 0.917. For the machine learning models, the sensitivity and specificity were as follows: logistic regression, 0.92 and 0.85; XGBoost, 0.90 and 0.85; decision trees, 0.88 and 0.86; and ensemble learning, 0.91 and 0.85, respectively. The resulting AUCs were, in the same order: 0.938, 0.937, 0.930, and 0.941, respectively.

**Discussion:**

In this proof-of-concept study, machine learning algorithms achieved improved diagnostic performance compared to the major/minor weighing system as used in the MDE2015 criteria. Moreover, these models provide quantifiable certainty levels of the diagnosis, potentially enhancing interpretability for clinicians. Additionally, they allow for easy incorporation of new and/or refined criteria, such as the individual weight of advanced imaging modalities such as CTA or [^18^F]FDG PET/CT. These promising preliminary findings warrant further studies for validation, ideally in a prospective cohort encompassing the full spectrum of patients with suspected IE.

**Supplementary Information:**

The online version contains supplementary material available at 10.1007/s00259-024-06774-y.

## Introduction

Infective endocarditis (IE) is a serious condition causing substantial morbidity and mortality and a well known potential complication of prosthetic valve implantation. The risk of developing IE after prosthetic valve implantation is estimated to be at least 0.4-1.0% per patient-year [1, 2]. Simultaneously, disease severity increases with prosthetic valves being present, with a reported in-hospital mortality rate of 19.9% [3]. A timely and accurate diagnosis of prosthetic valve endocarditis (PVE) is vital for determining the best treatment plan and patient outcome. However, the clinical presentation of PVE is often variable and non-specific, making it challenging to diagnose.

In 1994 the Duke criteria were introduced as a framework for diagnosing endocarditis and although they were primarily intended for use in epidemiologic studies, they have also been used extensively in clinical settings. Since their inception, the Duke criteria have undergone four major modifications [4–6]. The second modification was made by the European Society of Cardiology (ESC) in 2015 [5] and in 2023 two other updates were published: one by the ESC and another by the International Society of Cardiovascular Infectious Diseases (ISCVID) [6, 7]. The 2015 ESC and 2023 ISCVID and ESC modifications of the Duke criteria, among other changes, introduced advanced imaging modalities such as cardiac Computed Tomography Angiography (Cardiac CTA) and [^18^F]fluorodeoxyglucose positron emission tomography/computed tomography ([^18^F]FDG PET/CT) as major criteria, while leaving the original weighing system of major and minor criteria unchanged. This may have introduced bias to the endocarditis criteria. This is due to the fact that the diagnostic system applies weight to all imaging modalities as if they were echocardiography, even though multimodality imaging has additional diagnostic power over echocardiography alone. The risk of this is that findings from these powerful diagnostic tools are not valued correctly for the diagnosis of PVE. And while the Duke criteria and their modifications clearly state that they are not meant to replace clinical judgment, it is still beneficial to strive to eliminate sources of potential bias from the endocarditis criteria as much as possible.

Additionally, the original Duke criteria and their subsequent modifications all allow for a ‘possible’ endocarditis designation. In clinical practice a considerable number of patients receive this designation for the diagnosis, which limits the clinical utility of the criteria. The overall sensitivity and specificity of the modified Duke criteria for IE is approximately 80%, and for patients with prosthetic heart valves or cardiac implanted electronic devices this is likely lower [8]. Recent studies regarding the exact sensitivity, specificity of the modified Duke/ESC (MDE2015) criteria and the rate at which they result in an unclear or ‘possible’ diagnosis are scarce, and should be interpreted with caution [9].

Machine learning algorithms are increasingly recognized as valuable diagnostic tools in the medical field, with applications spanning a wide range of diagnostic and prognostic applications [10]. Decision tree models are an early form of machine learning, but they continue to be popular due to their interpretability and ease of implementation [11]. The Lasso logistic regression model, a variant of logistic regression that utilizes L1 regularization, has demonstrated its utility in various studies [12, 13]. XGBoost is another model that has garnered attention in recent years. It is an implementation of decision trees which uses gradient boosting to increase its predictive power and it has shown promise in predicting diverse medical conditions [14–16]. In fact, in a recent study, it has been shown that diagnosis of aortic PVE can be substantially enhanced by using a machine learning model that includes automated segmentation and radiomics for interpretation of PET/CT as improved major criterion for the ESC-2015 criteria. The improved performance positively impacted on overall diagnostic accuracy [17].

The aim of this study was to improve the diagnostic accuracy of the (MDE2015) criteria through application of machine learning algorithms, while using the same diagnostic features that also comprise the MDE2015 criteria. Instead of the traditional division between major and minor criteria, our approach was to weigh the different MDE2015 criteria in a data-driven manner, using the aforementioned machine learning algorithms. To the best of our knowledge, this is the first study using machine learning in order to optimize application of the MDE2015 criteria. The goal of this was assuring that the different features comprising the MDE2015 criteria were used in accordance with their predictive value for the diagnosis of PVE. We hypothesized that this approach would make better use of the multiple diagnostic advancements that have found their way into clinical practice for diagnosing PVE over the past decades, and therefore outperform the prediction system of major and minor criteria as used by the MDE2015 endocarditis criteria, both in terms of interpretability and diagnostic accuracy.

## Methods

For this study, data were used from an earlier published multicentre study [18]. In this study, prosthetic valve recipients from six cardiothoracic centres in the Netherlands were included if they had undergone [^18^F]FDG PET/CT imaging for suspected PVE. All clinical data and the outcomes of the imaging modalities that were included in the 2015 ESC guidelines (i.e. cardiac CTA and [^18^F]FDG PET/CT) were available, with the exception of vascular and immunologic phenomena, which were not always documented. All available clinical data was combined to calculate the clinical MDE2015 criteria for all study subjects. Histopathologic criteria were not used to predict the disease, but when available, they were used by the endocarditis team to establish the final diagnosis.

The original paper used the modified Duke criteria from prior to the ESC 2015 update, as proposed by Li et al. [4]. The reason for this was that this study aimed to evaluate the impact of [^18^F]FDG PET/CT on the diagnosis of PVE. Thus, incorporating findings of PET/CT into the Duke criteria carried a potential risk of bias at that stage. However, findings from PET/CT and CTA were available in the original dataset. When these imaging findings were incorporated in the MDE2015 criteria, 6 patients initially classified as negative were reclassified as “possible” PVE, and likewise 3 were reclassified from “possible” to “definite” PVE (Table 1).

**Table 1:** Patient demographics and clinical characteristics, by presence of PVE [18]

Demographics	PVE confirmed (*n = 80*)	PVE rejected (*n = 80*)	Total (*n = 160*)	*p*-value
Age, median [IQR], y	52 [35–68]	68 [53–75]	62 [43–73]	**< 0.001**
Sex (male), n (%)	49 (61.3)	59 (73.8)	108 (67.5)	0.128
BMI, mean ± SD, kg/m^2^	24.9 ± 5.4	25.6 ± 4.6	25.2 ± 5.0	0.392
Diabetes mellitus, n (%)	10 (13)	13 (16.3)	23 (14.4)	0.653
Prior history of IE, n (%)	*15* (19)	18 (23)	33 (20.6)	0.563
Multiple PV present, n (%)	9 (11.3)	12 (15)	21 (13.1)	0.640
CIED present, n (%) *(Missing data)*	8 (12.7) *(17 missing)*	9 (15.8) *(23 missing)*	17 (14.2) *(40 missing)*	0.794
Surgery performed, n (%)	44 (55)	1 (1.3)	45 (28.1)	**< 0.001**
MDE2015 Criteria n (%) Imaging (major) Blood cultures (major) Predisposition Fever Vascular phenomena Immunologic phenomena Microbiologic evidence (minor)	74 (92.5) 55 (68.8) 80 (100) 58 (72.5) 8 (10) 5 (6.3) 8 (10)	12 (15) 18 (17.5) 80 (100) 27 (33.8) 0 (0) 0 (0) 4 (5)	86 (54) 73 (46) 160 (100) 85 (53) 8 (5) 5 (3) 12 (7.5)	**< 0.001** **< 0.001** **< 0.001** **< 0.001** **0.007** 0.059 0.369
*MDE2015 classification, n (%)*				
Rejected Possible Definitive	3 (3.8) 18 (22.5) 59 (73.8)	48 (60) 30 (37.5) 2 (2.5)	51 (31.9) 48 (30) 61 (38.1)	**< 0.001**
Mortality in follow-up, n (%)	10 (12.5)	11(13.8)	21 (13.1)	1.000

*Legend %* percentage of patient population, CIED: cardiac implanted electronic devices, IQR: interquartile range, n: number of patients, MDE2015: modified Duke criteria according to the 2015 European Society of Cardiology guideline [5], PV: prosthetic valve, PVE: prosthetic valve endocarditis, SD: standard deviation, y: years

The algorithms for data-driven prediction of PVE were written in Python 3.9® [Python Software Foundation, DE, USA]. To minimize the risk of overfitting the predictive models, internal cross-validation was performed using a repeated stratified K-fold to divide the data in training and test sets, using 4 folds per iteration, repeated for a total of 20 times. A Lasso logistic regression, a decision tree model and a gradient boosted decision tree model (XGBoost) were used for predicting PVE using the MDE2015 features. Additionally, these models were combined in an ensemble learning model to give one overall prediction of the disease. The maximum number of iterations to reach convergence was set to 1000. For XGboost and the decision tree classifier, the maximum depth of the trees was set to 4. For the ensemble learning classifier, the probability of PVE presence according to all the other machine learning models was averaged without first dichotomizing these predictions. Additionally, a single run of K-fold was performed to allow for a tentative statistical comparison between the models and the MDE2015 criteria. This analysis focused on the subgroup of the patient population with a possible endocarditis according to the MDE2015 criteria. For all models, a probability cut-off of 0.5 was used to classify a patient as having confirmed or rejected PVE.

The performance of the models based on their binary predictions of disease presence or absence across the repeated K-fold iterations was used for calculating their averaged performance metrics. We included accuracy, precision, F1-score, sensitivity, and specificity. All aforementioned algorithms give a probabilistic prediction for the presence of PVE. This probability-per-case prediction was also averaged over the 20 repetitions of K-fold and this was used for the Receiver Operating Curve (ROC) and Area Under the Curve (AUC) analysis curves.

The overall predictive performance of the machine learning models was compared to the prediction of the disease according to the MDE2015 criteria as described in the 2015 ESC guidelines [5]. As previously reported, the final diagnosis of PVE in this cohort was based on multidisciplinary consensus from the endocarditis team, using all the available clinical data including surgical findings whenever performed and a minimum of 1 year of follow-up [18]. This was used as the gold standard for the diagnosis.

The MDE2015 criteria allow for a ‘possible’ designation for IE. This presents a challenge whenever they are compared to any predictors with a dichotomous outcome, as the MDE2015 criteria do not make a definitive prediction of the disease’s presence or absence for ‘possible’ cases. We addressed this by calculating the MDE2015 criteria diagnostic accuracy in four scenarios: (1) assuming a 50% prediction rate for ‘possible’ cases, both for those with and without the disease; (2) treating all ‘possible’ cases as positive, (3) treating all ‘possible’ cases as negative; and (4) excluding all ‘possible’ cases. We also drew a ROC-curve and calculated the corresponding AUC for the MDE2015 criteria using scenario’s 2 and 3 to give an visualization of their performance.

Next, we conducted a feature importance analysis for all machine learning models, with exception for the ensemble learning model, since ensemble learning does not allow for this type of analysis. We used feature importance analysis to get a rough estimate of the weight the models attributed to the different features that make up the MDE2015 criteria.

## Statistics

Demographic data are represented as means +/- SD for continuous variables if they were normally distributed and as median and interquartile range (IQR) if they were not normally distributed. Categorical variables are shown as frequencies. Demographic differences across the outcome variable of presence of PVE by final diagnosis were tested for statistical significance using Student’s t-test or Mann-Whitney U test, depending on normality or non-normality of the data. Fischer’s exact test was used for comparing categorical variables. The single run of K-fold cross-validation using four folds was also performed to test for demographic differences across the folds. This analysis was added as supplemental data for illustrative purposes, showing that K-fold cross-validation results in balanced groups.

ROC curves were obtained for both the machine learning models and the MDE2015 criteria, and they were utilized for AUC analysis. In repeated K-fold cross-validation the AUCs obtained across the repeated folds cannot directly be compared with the AUC achieved by using the MDE2015 criteria due to the interdependence of the different ROCs that were obtained through this method. Therefore no further statistical analyses were performed, and the performance of the models was added using descriptive statistics only.

A two-tailed P-value of *≤* 0.05 was considered statistically significant in all analyses. Statistical analysis was performed using IBM® SPSS 26 [IBM Corp, NY, USA].

## Results

As previously reported, 160 patients with suspicion of PVE, were included in the study [18]. PVE was established as the final diagnosis by the endocarditis team in 80 (50%) of these patients and rejected in the others. A full overview of patient characteristics, stratified by their assigned K-fold group in the cross-validation, is shown in Table 1.

### MDE2015 criteria

**Fig. 1:**
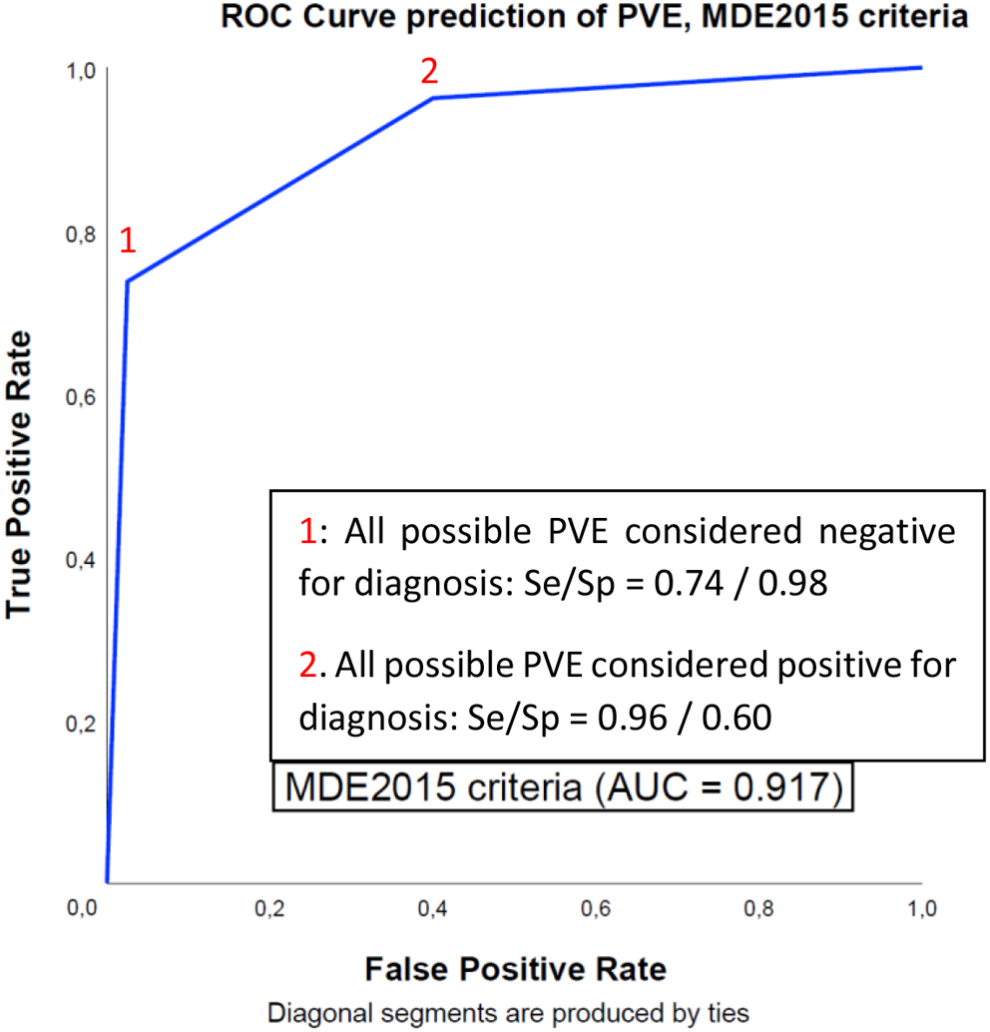
ROC curves probability prediction of PVE, Modified Duke/ESC 2015 criteria. This figure provides a visualisation of the performance of the Modified Duke / ESC 2015 criteria in two different scenarios as described above. AUC: Area under the curve, ESC: European Society of Cardiology, MDE2015: Modified Duke criteria according tothe modification as proposed by the ESC in 2015, PVE: prosthetic valve endocarditis, ROC: Receiver Operating Curve, Se: Sensitivity, Sp: Specificity

In this cohort, 48 patients (30%) were classified as ‘possible’ endocarditis according to the MDE2015 criteria. When the predictions in this group were considered as correct in 50% of cases, both for those with and without the disease, sensitivity and specificity were 0.85 and 0.79, respectively. When all patients with ‘possible’ endocarditis were considered as confirmed cases, sensitivity and specificity of the criteria were 0.96 and 0.60, respectively. When they were designated as rejected endocarditis, this resulted in a sensitivity and specificity of 0.74 and 0.98, respectively. These two scenarios (all possible endocarditis considered positive/negative) were combined for ROC analysis. This yielded an AUC of 0.917, see also Fig. 1. When only patients with either a rejected or definite diagnosis of endocarditis were considered, the MDE2015 criteria achieved a sensitivity and specificity of 0.95 and 0.96, respectively.

### Machine learning models

**Fig. 2:**
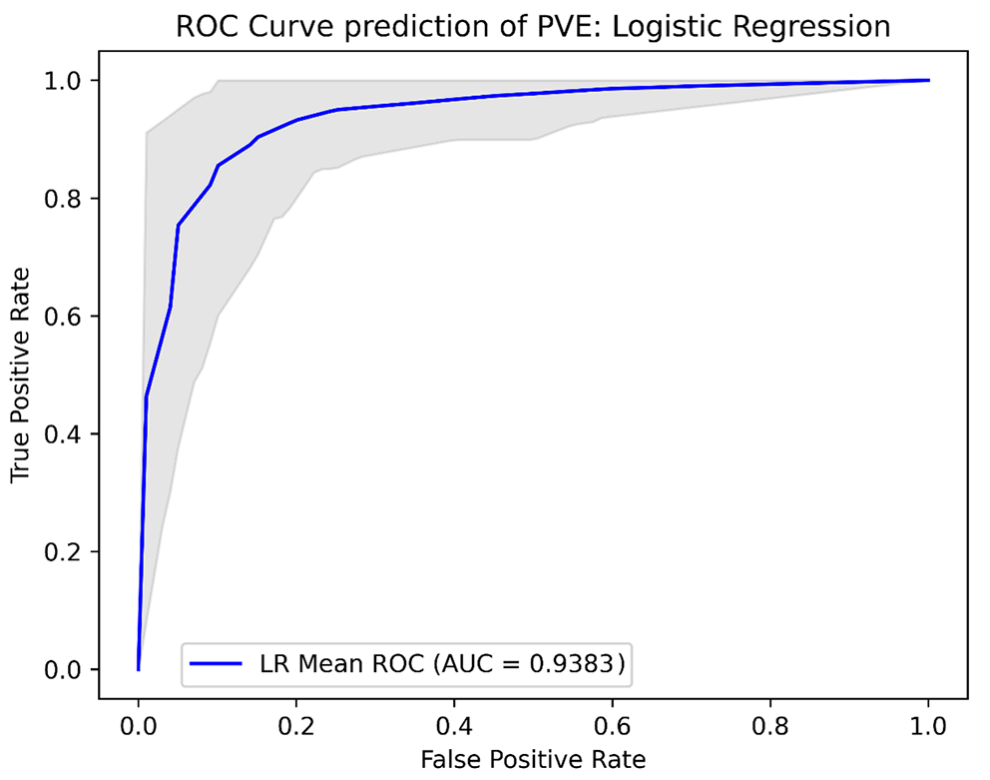
ROC curves probability prediction of PVE, Logistic Regression. ROC analysis of the lasso logistic regression model, validated using repeated K-fold cross-validation. The blue line indicates the mean ROC curve across 100 iterations in repeated K-fold. The shaded area indicates the 95% confidence interval, illustrating the range of sensitivity and specificity combinations that could be encountered when applying this machine learning model across various threshold values for predicting PVE. AUC: Area under the curve, LR: logistic regression, PVE: prosthetic valve endocarditis, ROC: Receiver Operating Curve

**Fig. 3:**
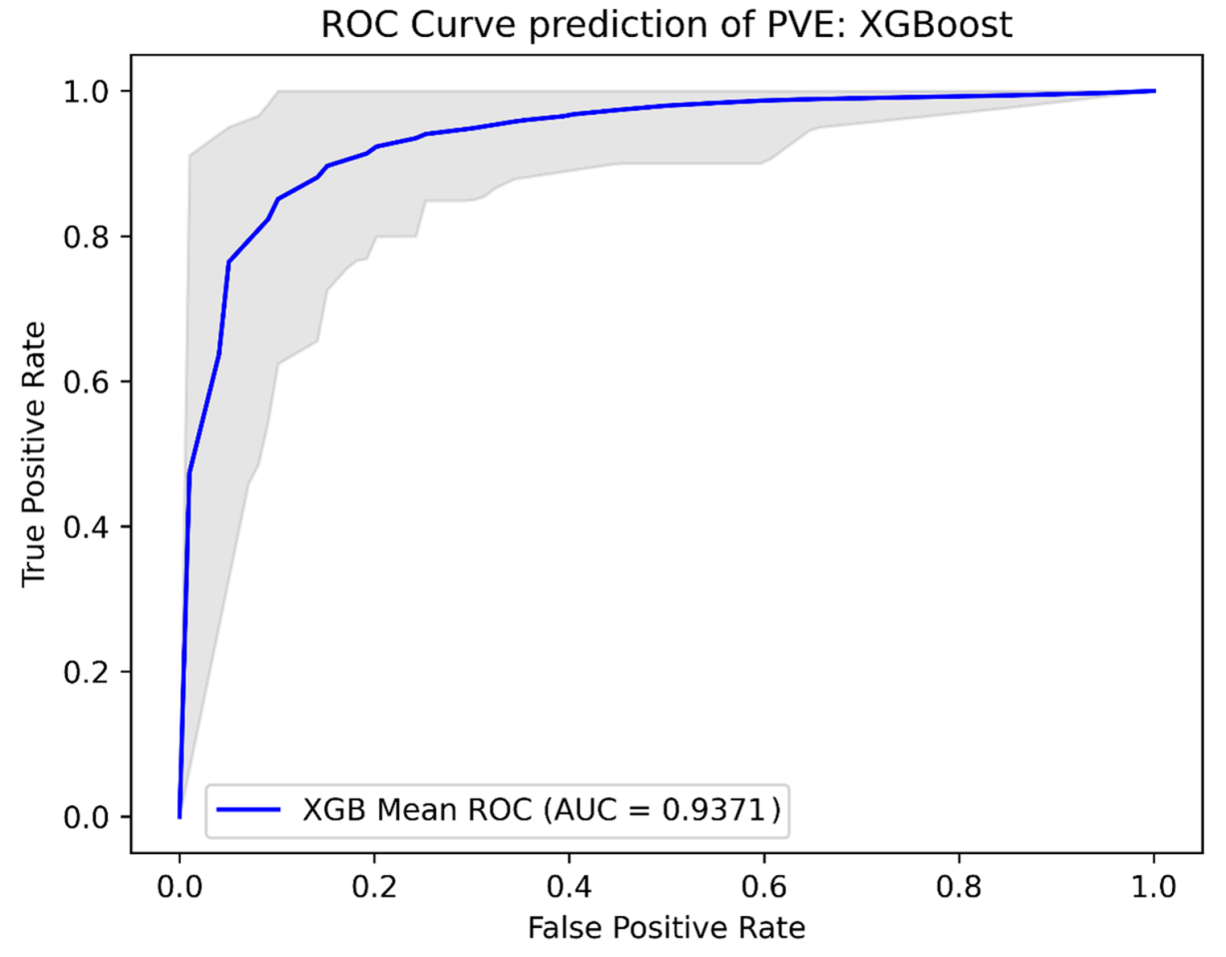
ROC curves: XGBoost ROC analysis of the XGBoost model, validated using repeated K-fold cross-validation. The blue line indicates the mean ROC curve across the 100 iterations in repeated K-fold. The shaded area indicates the 95% confidence interval, illustrating the range of sensitivity and specificity combinations that could be encountered when applying this machine learning model across various threshold values for predicting PVE. AUC: Area under the curve, XGB: XGBoost, PVE: prosthetic valve endocarditis, ROC: Receiver Operating Curve

**Fig. 4:**
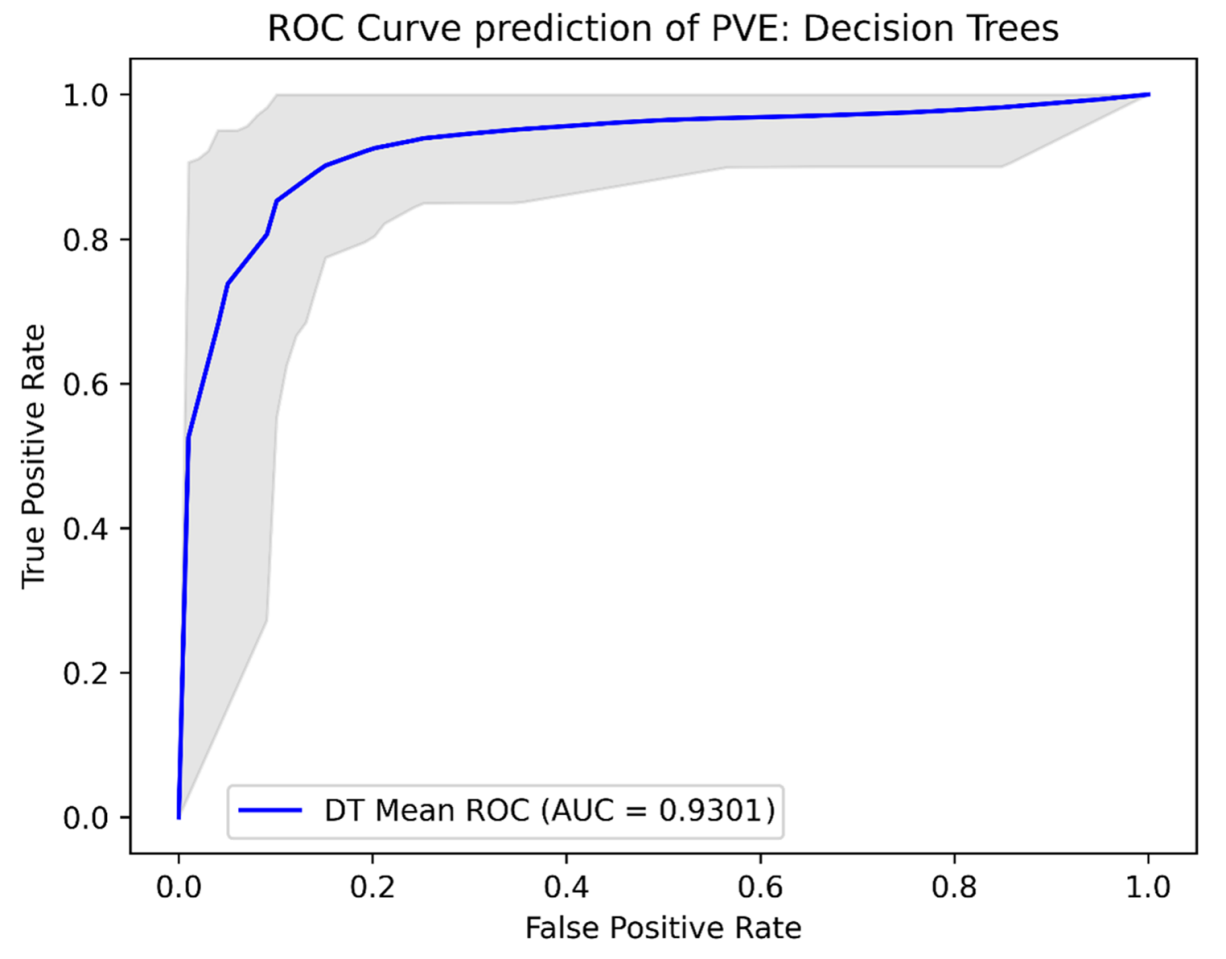
ROC curves probability prediction of PVE, Decision Trees Legend ROC analysis for the decision trees model, validated using repeated K-fold cross-validation. The blue line indicates the mean ROC curve across the 100 iterations in repeated K-fold. The shaded area indicates the 95% confidence interval, illustrating the range of sensitivity and specificity combinations that could be encountered when applying this machine learning model across various threshold values for predicting PVE. AUC: Area under the curve, DT: decision trees, PVE: prosthetic valve endocarditis, ROC: Receiver Operating Curve

**Fig. 5:**
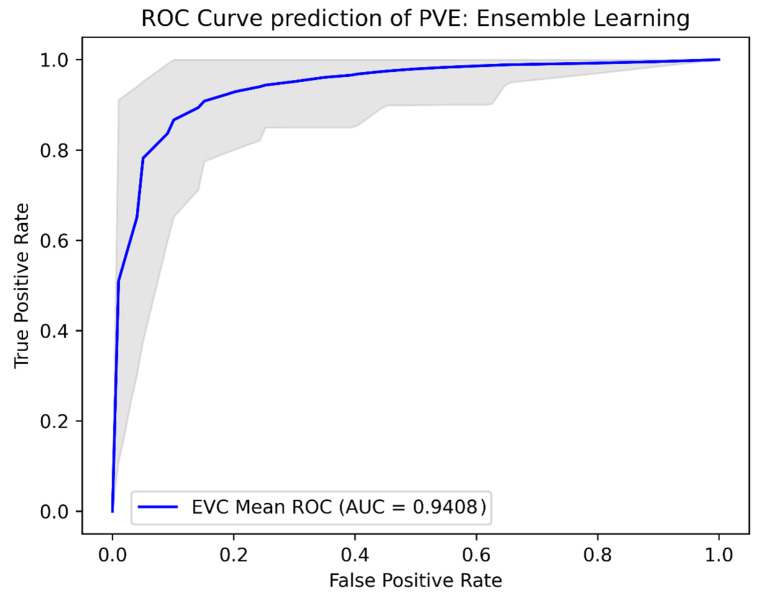
ROC curves probability prediction of PVE, Ensemble Learning Legend: ROC analysis for the ensemble learning model, validated using repeated K-fold cross-validation. The blue line indicates the mean ROC curve across the 100 iterations in repeated K-fold. The shaded area indicates the 95% confidence interval, illustrating the range of sensitivity and specificity combinations that could be encountered when applying this machine learning model across various threshold values for predicting PVE. AUC: Area under the curve, EVC: ensemble learning, PVE: prosthetic valve endocarditis, ROC: Receiver Operating Curve

The Lasso Logistic Regression model achieved a sensitivity and specificity of 0.93 and 0.85, respectively. The XGboost model obtained a sensitivity and specificity of 0.90 and 0.85, respectively. The decision tree classifier had a sensitivity and specificity of 0.88 and 0.86, respectively, while the ensemble learning model achieved a sensitivity and specificity of 0.91 and 0.85, respectively. The AUCs of the models were 0.938 for the Lasso Logistic Regression model, 0.937 for the XGBoost model, 0.930 for the decision tree model, and 0.941 for the ensemble Learning model. Further performance metrics are shown in Table 2 and the ROC-curves for the models are shown in Figs. 2, 3, 4 and 5.

**Table 2:** Machine learning models performance metrics, across repeated K-fold

Metrics	Logistic regression	XGBoost	Decision trees	Ensemble learning
Accuracy, median [IQR]	0.88 [0.85-0.93]	0.88 [0.85-0.93]	0.88 [0.84-0.90]	0.88 [0.82-0.90]
Presicion, median [IQR]	0.86 [0.82-0.90]	0.86 [0.82-0.90]	0.86 [0.82-0.91]	0.86 [0.82-0.91]
F1-score, median [IQR]	0.89 [0.86-0.93]	0.88 [0.86-0.92]	0.87 [0.83-0.91]	0.89 [0.86-0.93]
Sensitivity, mean±SD	0.93±0.05	0.90±0.09	0.88±0.11	0.91±0.08
Specificity, mean±SD	0.85±0.07	0.85±0.07	0.86±0.08	0.85±0.07
AUC, mean±SD	0.94±0.03	0.94±0.04	0.93±0.04	0.94±0.03

*Legend* IQR: interquartile range, SD: standard deviation

There were substantial differences between the predictive models in how much weight was attributed to the different features comprising the MDE2015 criteria. Notably, all machine learning models attributed a large weight to (advanced) imaging modalities. With the Lasso logistic regression, the coefficient of imaging was 3.5, more than 3 times higher than that of major blood cultures. In the XGboost model, the weight of imaging was 0.91 while for the decision tree it was 0.75. An overview of feature weight per model is shown in Table 3.

**Table 3:** Feature importance per predictive model (Repeated stratified K-fold)

Diagnostic model ^#^	Feature importance per MDE2015 criterium^1^					
	Blood cultures (major)	Imaging	Fever	Vascular phenomena*	Immunologic Phenomena*	Microbiologic evidence (minor)
Lasso logistic regression	1.015	3.506	0.644	0.348	0.167	0.211
XGBoost	0.052	0.906	0.023	0.000	0.000	0.020
Decision trees	0.092	0.746	0.040	0.037	0.034	0.050

*Legend*^1^Predisposition was not included in this table as a feature, since this was present in all patients

# Feature importance is measured differently in the various machine leaning models. For Logistic regression this represents the coefficients for the included features, for XGBoost it indicates total gain, while for decision trees it described the effect of a feature on the entropy in the model

* indicates features for which data was frequently missing

In the sub-analysis of a single run K-fold, focused specifically on those patients classified as ‘possible’ endocarditis, the models all achieved a sensitivity and specificity of 0.94 and 0.67, respectively. The AUCs and 95% confidence intervals of the models were 0.80 (0.67–0.93) for the Lasso Logistic regression, 0.82 (0.70–0.94) for the XGBoost model, 0.80 (0.67–0.93) for the Decision Tree model and 0.81 (0.69–0.94) for the Ensemble Learning model. Tested against an AUC of 0.50, the models obtained p-values of 0.001, < 0.001, 0.001 and < 0.001, respectively. See also supplemental data (Fig. 1).

## Discussion

This proof-of-concept study demonstrated the feasibility of machine learning models in prediction PVE with a potentially superior diagnostic accuracy compared to the MDE2015 criteria, while using the same features. Moreover, these models offer the possibility to quantify the level of uncertainty per prediction, enhancing clinical interpretability. Furthermore, they allow for easy incorporation of new and/or refined features, which would ensure that criteria based on machine learning models could easily be updated to keep them aligned with clinical practice as new and refined diagnostic tools become available. All machine learning models demonstrated similar performance in terms of binary predictions. There were small differences between the models in this regard, but these are unlikely to be of clinical relevance.

Performance of the MDE2015 criteria in the current study was comparable, if not superior relative to its performance in other recent publications. In one study the MDE2015 criteria achieved an AUC of 0.87 [8] compared to 0.917 in the current study. In another recent study in which ‘possible’ cases were classified as negative, a sensitivity and specificity of 0.84 and 0.71 were reported [9]. In our study, the application of the MDE2015 criteria yielded a sensitivity of 0.74 and a notably higher specificity of 0.98. This illustrates the robust performance of the machine learning models, as they were benchmarked against well-established diagnostic criteria showing good performance.

Interestingly, both the XGBoost and decision tree model assigned considerable weight to the outcome of imaging while downplaying the role of blood cultures, even though the latter are also a key component in the diagnosis of PVE. In the logistic regression model, the pronounced role of imaging was also present. Here the major blood cultures contribution to the final prediction of the model was higher, but still the coefficient for imaging was more than 3 times higher than that of blood cultures. The reasons for this could be the time points of the respective features in the diagnostic process. Blood cultures are frequently the reason for suspicion of IE in patients with prosthetic valves, while imaging is applied at a later stage in the process for specific evidence of the infection intracardially. The high weight attributed to imaging could also be due to the increasing accuracy of these modalities in detecting PVE, since in the ESC guidelines from 2015 to 2023, imaging consists of findings of not only echocardiography, but also that of cardiac CTA, [^18^F]FDG PET/CT and leucocyte scintigraphy. Simultaneously, it might also in part reflect a high reliance of participating endocarditis teams on imaging findings for their final diagnosis, implying a level of incorporation bias.

When interpreting feature importance analysis, it is also important to stress the differences between the models. In decision tree models and XGBoost, each feature is used sequentially to make a prediction. This means that feature importance signifies the contribution of these factors to the performance of the model (e.g. total gain for XGBoost and entropy for the decision trees). In contrast, Lasso logistic regression uses all features simultaneously. In this instance, feature importance shows how heavily the different factors influence the predicted probability of disease presence in the optimized model.

Some limitations of the current study need to be addressed. For this study we used a retrospective cohort primarily investigated for the value of [^18^F]FDG PET/CT. Given that the majority of patients were evaluated in tertiary care centres and all underwent [^18^F]FDG PET/CT our results may not generalize to wider clinical practice. Also, our data was collected around the time of the ESC modification and therefore, could not be adapted to the recent ISCVID or 2023 ESC modifications to the Duke criteria. Furthermore, as reported in the 2018 study [18] data on vascular and immunologic phenomena were often missing in the study cohort, which means the feature weights as found in our results can illustrate how these models function in this particular cohort, but they should not be generalized to general clinical practice. Despite these limitations, the findings of this study suggest that machine learning models could enhance the utilization of available information beyond what is possible with the MDE2015 criteria.

Several considerations need to be addressed before integrating these models into clinical practice. In infective endocarditis, obtaining a definitive diagnosis is challenging if not impossible unless surgery is performed. This necessitates reliance on team consensus and follow-up. A potential concern of that is that this process could lead to bias from the participating endocarditis teams inadvertently being incorporated into the machine learning models. Moreover, key diagnostic features may be underestimated according to the models, depending on their place in the diagnostic process and their prevalence in the target population.

Medical team consensus is also often more nuanced than a binary disease presence statement. Therefore, including a level of confidence in the diagnosis could provide a more accurate benchmark for evaluating the models. Similarly, it could be beneficial to document the level of confidence associated with the different clinical signs and diagnostic tools used in patient evaluation, as these factors are not standardized: e.g., image quality may affect results of imaging modalities, antibiotic (pre-) treatment can impact results, and clinical signs may not hold the same predictive value if these are linked to a pre-existing condition. Addressing these considerations could help minimize the repeated rounding errors caused by the digitization of these diagnostic features, which would lead to more transparent and objective diagnostic decisions.

For future validation, these models should be tested on a diverse patient cohort across various healthcare settings and ideally including those with native valves, prosthetic valves and implanted cardiac devices. Hyperparameter tuning would be important for these validation studies and might show performance differences between the machine learning models used. Given a sufficiently large and diverse patient cohort to avoid overfitting, these models could also be further refined with additional predictors to provide a more nuanced understanding of the added risk of IE associated with specific types of predisposition, clinical signs or findings of the various available diagnostic modalities.

## Conclusion

In this proof-of-concept study, machine learning algorithms demonstrated potentially superior performance in predicting PVE compared to the MDE2015 criteria, while using the same diagnostic features. Preliminary statistical tests were particularly promising for those with possible endocarditis according to the MDE2015 criteria. These results imply that the use of machine learning algorithms could potentially lead to improved diagnostic accuracy and interpretability for this challenging diagnosis. Future prospective validation studies are warranted to affirm whether these promising findings reflect a statistically significant improvement over the MDE criteria as they are currently used. Ideally, such studies would encompass the full spectrum of patients with suspected IE.

## Electronic supplementary material

Below is the link to the electronic supplementary material.

Supplementary Material 1

## Data Availability

The data are exclusively available for the purpose of reproducing the study results upon reasonable request [18].
