# Supplementary material for: Using machine learning to improve the diagnostic accuracy of the modified Duke/ESC 2015 criteria in patients with suspected prosthetic valve endocarditis – a proof of concept study
Source: Eur J Nucl Med Mol Imaging. 2024 Jun 21;51(13):3924–33. doi: 10.1007/s00259-024-06774-y (PMC11527948; doi:10.1007/s00259-024-06774-y)

# Supplemental data

**Table 1. Demographic data of a single run of K-fold cross-validation**

| *Demographics* | *Fold 1*  *(n=40)* | | *Fold 2*  *(n=40)* | | *Fold 3*  *(n=40)* | | *Fold 4 (n=40)* | | *Total (n=160)* | | *p-value* |
| --- | --- | --- | --- | --- | --- | --- | --- | --- | --- | --- | --- |
| Age, median [IQR],  y | 66 [47-72] | | 56 [39-73] | | 63 [37-73] | | 64 [51-76] | | 62 [43-73] | | 0.396 |
| Sex (male), n (%) | 30 (75.0) | | 28 (70.0) | | 30 (75.0) | | 20 (50.0) | | 108 (67.5) | | 0.053 |
| BMI, mean ± SD,   kg/m^2^ | 25.8 ± 4.4 | | 25.0 ± 4.9 | | 24.3 ± 5.2 | | 25.9 ± 5.5 | | 25.2 ± 5.0 | | 0.434 |
| Diabetes Mellitus,   n (%) | 8 (20.0) | | 8 (20.0) | | 3 (7.5) | | 4 (10.0) | | 23 (14.4) | | 0.242 |
| Prior history of IE,   n (%) | 7 (17.5) | | *5 (12.5)* | | *10 (25.0)* | | *11 (28.2)*  *1 missing data* | | 33 (20.6)  *1 missing data* | | 0.302 |
| Multiple PV  present, n (%) | 4 (10.0) | | 8 (20.0) | | 4 (10.0) | | 5 (12.5) | | 21 (13.1) | | 0.502 |
| CIED present, n (%) | *5 (16.7)*  *10 missing* | | *4 (11.8)*  *6 missing* | | *5 (19.2)*  *14 missing* | | *3 (10.0)*  *10 missing* | | 17 (14.2)  *40 missing* | | 0.734 |
| Surgery performed,  n (%) | 14 (35.0) | | 9 (22.5) | | 10 (25.0) | | 12 (30.0) | | 45 (28.1) | | 0.612 |
| MDE classification,  n (%)  Rejected  Possible  Definite | 14 (35)  11 (27.5)  15 (37.5) | | 10 (25.0)  15 (37.5)  15 (37.5) | | 16 (40.0)  8 (20.0)  16 (40.0) | | 10 (25.0)  15 (37.5)  15 (37.5) | | 51 (31.8)  48 (30.0)  61 (38.1) | | 0.885 |
| MDE Criteria, n (%)  Imaging (major)  Blood cultures (major)  Predisposition  Fever  Vascular   phenomena  Immunologic  phenomena  Microbiologic  evidence (minor) | 22 (55)  18 (45)  40 (100)  22 (55)  1 (2.5)  1 (2.5)  2 (5) | | 22 (55)  19 (47.5)  40 (100)  24 (60)  2 (5)  1 (2.5)  5 (12.5) | | 21 (52.5)  15 (37.5)  40 (100)  18 (45)  1 (2.5)  0 (0)  3 (7.5) | | 21 (52.5)  21 (52.5)  40 (100)  21 (52.5)  4 (10)  3 (7.5)  2 (5) | | 86 (54)  73 (46)  160 (100)  85 (53)  8 (5)  5 (3)  12 (7.5) | | 0.992  0.596  1.000  0.597  0.368  0.270  0.539 |
| Final diagnosis PVE,  n (%) | 20 (50) | | 20 (50.0) | | 20 (50.0) | | 20 (50.0) | | 80 (50.0) | | 1.000 |
| *Final diagnosis PVE*  *Grouped by MDE  class* | ***Yes*** | ***No*** | ***Yes*** | ***No*** | ***Yes*** | ***No*** | ***Yes*** | ***No*** | ***Yes*** | ***No*** |  |
| Rejected  Possible  Definite | 1  4  15 | 13  17  0 | 0  6  14 | 11  9  1 | 1  4  15 | 16  4  1 | 1  4  15 | 11  10  0 | 3  18  59 | 48  30  2 | 0.832  0.788  0.571 |
| Mortality in follow-  up, n (%) | 3 (7.5) | | 4 (10.0) | | 7 (17.5) | | 7 (17.5) | | 21 (13.1) | | 0.427 |

**Figure 1. ROC curves for the prediction of PVE for patients with possible endocarditis according to the MDE2015 criteria. Model comparisons are based on a single run of K-fold**


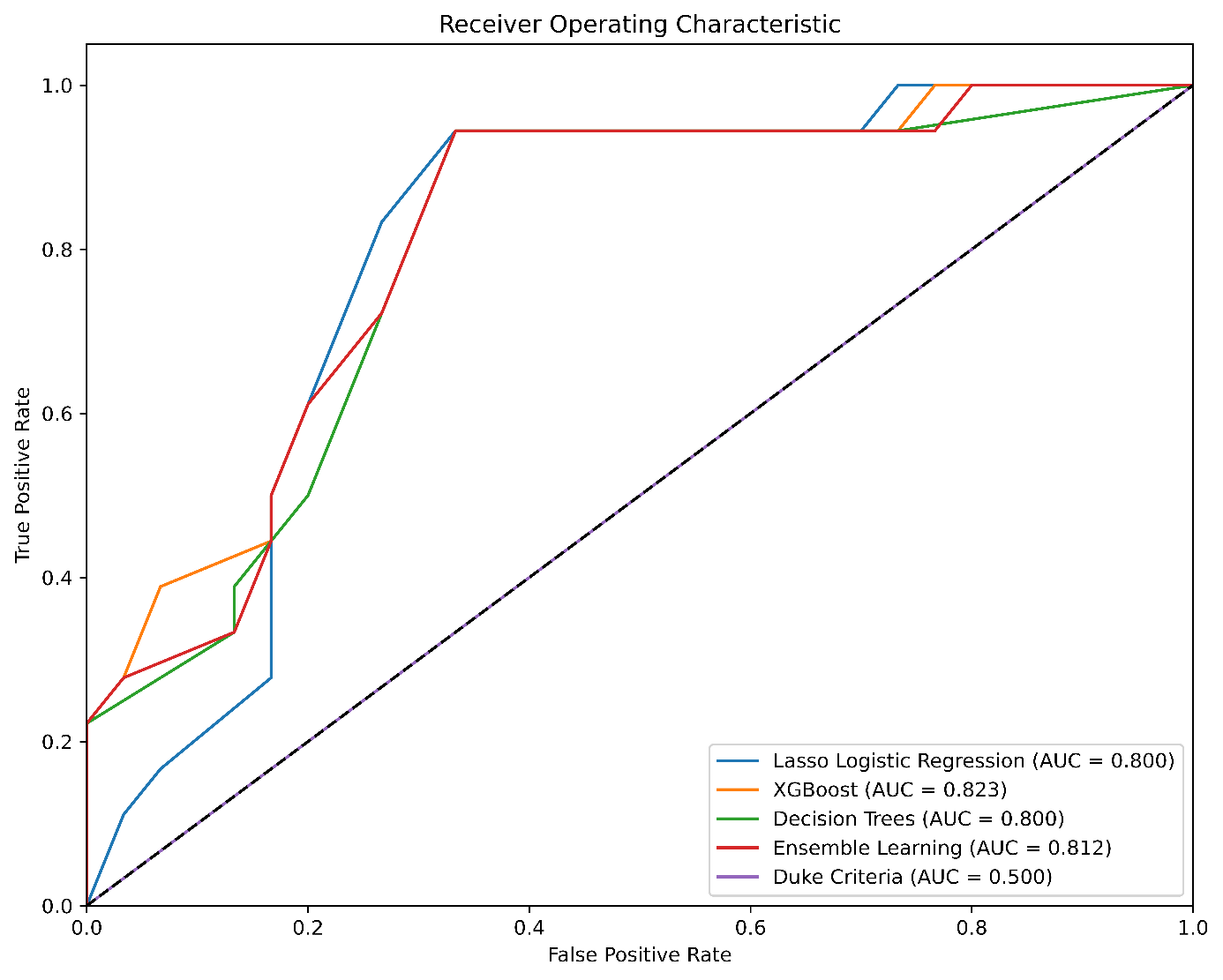

Supplement: Supplementary file 1 — Supplementary Material 1 [file 259_2024_6774_MOESM1_ESM.docx]
